# Patients' experiences of receiving hospital care from a mobile care unit – a qualitative interview study

**DOI:** 10.1080/17482631.2026.2626446

**Published:** 2026-02-07

**Authors:** Christofer Teske, Ghassan Mourad, Micha Milovanovic

**Affiliations:** a Department of Health, Medicine, and Caring Sciences, Linköping University, Linköping, Sweden; b Department of Emergency Medicine in Norrköping and Department of Biomedical and Clinical Sciences, Linköping University, Linköping, Sweden

**Keywords:** Hospital at home, mobile care, mobile care unit, patients, qualitative interviews

## Abstract

**Introduction:**

Demographic changes have increased the number of older adults with chronic diseases, leading to more emergency visits and poorer outcomes. In response, Sweden’s “Close care” initiative promotes patient-centered care through models such as mobile care units, which provide hospital care in patients’ homes during office hours. To support their integration, this study explored patients’ experiences of receiving care from mobile care units in Sweden.

**Method:**

A qualitative interview design was used. Data were collected through individual interviews with 17 patients receiving care from mobile care units in different regions of Sweden between June 2021 and May 2025. Interviews were transcribed verbatim and analyzed using manifest conventional content analysis.

**Result:**

The findings emerged into two main categories with six subcategories: “The home as a care setting” and “Comprehensive patient-centered care”. Patients valued mobile care for its comfort, reduced stress, and decreased need for hospital visits, preferring it for mild conditions while favoring hospital care for acute illness.

**Conclusion:**

Patients appreciated the person-centered, home-based care, which reduced logistical challenges and supported daily routines and independence. However, some questioned its suitability for severe conditions, preferring hospital care for its advanced resources and 24/7 availability.

## Introduction

Over the past decades, demographics have shifted, leading to an increase in the number of older people in most countries, a trend that is projected to continue (Nilsson, 2022; Population, 2012; WHO, 2024). With aging populations, there is also a rising prevalence of chronic diseases, as older people are more likely to experience multiple chronic diseases, resulting in increased annual emergency care visits (Pines et al., 2013). The combination of increased emergency care visits, crowding and limited bed availability in the hospital departments are linked to a higher incidence of medical errors, negative patient experiences, and worse outcomes, including mortality (Af Ugglas et al., 2020; Ekermo et al., 2023; Epstein et al., 2012; McCusker et al., 2014). Furthermore, the life circumstances of older people worldwide are highly diverse and closely tied to socio-economic factors, which significantly influence their health outcomes and access to care (Atella et al., 2019; WHO, 2024). As chronic diseases become more prevalent and the need for advanced care increases, there is a growing interest in exploring alternative care models outside traditional hospitalisation. These models could enhance clinical outcomes, reduce societal costs and above all increase patient satisfaction (Jessup et al., 2020).

The Swedish care system is currently undergoing a transformation with the transition to “Close care” by decision of the Swedish government. This initiative aims to create a more patient-centred care where primary care becomes the foundation of the healthcare system (Regioner, 2023; Socialdepartementet, 2023). Shifting the focus from hospital-based care to patient-centred care aligns with international research exploring alternatives to traditional hospital care for elderly patients with chronic illnesses. By providing hospital care at home or in local health centres, healthcare can become more accessible and tailored to individual needs (Cardona-Morrell et al., 2017; Lin et al., 2024; Shepperd et al., 2016).

Mobile care is a form of hospital care delivered to the patients’ home. In literature, mobile care is referred to as Hospital at Home (Monalto, 2023), Early Discharge Units (Parsons et al., 2017; Williams et al., 2022), Geriatric Assessment Unit (Nikolaus et al., 1999) and Mobile Care Unit (Teske et al., 2023). Mobile care refers to care provided on site where patients are located in order to reduce hospital admissions and improve continuity of care (Monalto, 2023; Nikolaus et al., 1999; Teske et al., 2023). However, this type of care is provided only during office hours, in contrast to hospital at home that is available around the clock. Patients who typically require admission to a hospital ward receive monitoring, in-person care, diagnostic services (such as lab tests, ECGs, and radiography), and intravenous medications at home mainly from registered nurses and physicians (Teske et al., 2024). This approach has been suggested as a substitute for routine hospitalisation in patients with acute conditions and is supported by research across various patient groups and countries (Caplan et al., 2012; Conley et al., 2016; Edgar et al., 2024; Gonçalves-Bradley et al., 2017). The mobile care units in our study focus on frail older adults, defined as individuals over 65 years of age with multiple chronic conditions and extensive needs for both outpatient and inpatient medical care, according to the definition by the National Board of Health and Welfare in Sweden (Socialstyrelsen, 2011). The patient's may be referred to a mobile care unit from primary care or the hospital (Socialstyrelsen, 2011). The physician in the mobile care unit then determines whether the patient should be part of the unit or not. The patient's health status is monitored by home care providers, or healthcare professionals.

To date, research conducted on mobile care units has been predominantly quantitative in nature and has revealed positive outcomes in terms of patient and carer satisfaction (Caplan et al., 2012; Chair et al., 2024; Edgar et al., 2024). However, there is a lack of studies that explore patients' experiences with mobile care. The present study aims to contribute in-depth knowledge about how care through mobile care units can complement traditional care provided at hospitals. In doing so, the study may also serve as a basis for formulating relevant objectives in the transition toward “close care”.

## Method

### Design

We adopted a qualitative, inductive approach, using the content analysis methodology described by Hsieh and Shannon (2005). Individual interviews were chosen because our study participants were old and frail but also were recruited from multiple locations across the country. Transporting participants to joint sessions, such as focus groups, would have been ethically and logistically inappropriate. This method involved deriving codes and themes directly from the data. The main goal of this design was to identify, analyse, and categorise the qualitative data. This qualitative approach was selected because it enabled an in-depth understanding of patients' subjective experiences, which would not have been accessible through quantitative methods. The study adhered to the Standards for Reporting Qualitative Research (SRQR). and received approval from the Ethics Review Authority in Uppsala, Sweden (registration number: 2020-06986), dated 2021-02-19.

### Sample and setting

Between June 2021 and May 2025, interviews were conducted with patients from four different mobile care units in Sweden. These units are each located in different regions in cities with populations ranging from 61,000 to 160,000. These units offered hospital care at home, with the number of home care patient numbers ranging from 5 to 15. The invitation to participate in the study was issued by either the department head or a senior supervising physician at the healthcare facility. The participants were recruited by a registered nurse working in the unit. To be included, patients needed to be affiliated with the mobile care unit, able to understand Swedish, and to converse over the phone without hearing loss or cognitive impairments. Out of 19 invited patients, 17 agreed to participate. Participant characteristics are detailed in Table I.

**Table I.** Demographic characteristics of participants.

	Patients
**Gender -** male/female	7/10
**Disease “classification”**	
COPD	4
Chest pain	2
Heart failure	3
Chronic ulcer	2
Chronic pain	1
Non-specific/multimorbidity	2
Pneumonia	1
Adverse drug reaction	2

### Data collection and procedure

Patients who consented to participate in the study were provided with both verbal and written information by a registered nurse affiliated with the mobile care unit. They were assured of confidentiality, with data access restricted to the research team in accordance with the ethical guidelines of the Swedish Research Council. Subsequently, the first author (CT) contacted each participant individually. During this initial contact, CT introduced himself, outlined the study’s purpose and procedures, and emphasised the participants’ right to withdraw at any time without providing a reason. Participants were also invited to ask questions or request additional information prior to the interview. If the participant was available, the interview was conducted immediately; otherwise, a new appointment was scheduled. Data collection was conducted through individual telephone interviews (*n* = 17) using a semi-structured interview guide. This guide, collaboratively developed by all authors, comprised open-ended questions and was supplemented with follow-up probes to elicit more detailed responses (see Table II). A pilot interview was conducted prior to the main data collection. As no revisions to the guide were deemed necessary, the pilot interview was included in the final analysis. All interviews were conducted by CT, a registered nurse in emergency care with prior experience in qualitative interviewing and no previous care relationship with the participants. The interview began with participants introducing themselves and sharing their experiences of receiving care from a mobile care unit. They were encouraged to speak openly, with occasional prompts for clarifications or further details. The interviews lasted between 18 and 35 minutes, except for one that lasted 12 minutes. These were audio-recorded and transcribed verbatim.

**Table II.** Interviewguide.

Tell me about what happened the last time you became acutely ill. How did you contact healthcare? What expectations did you have when you contacted healthcare? What help were you offered when you contacted healthcare? How did you experience the care you received? … depending on whether it was via ambulance, emergency department, or hospital ward. What opportunities did you have to influence the care that was offered? How often do they come? Can you influence them when they come? Would you have wished to be treated in a different way? How do you experience receiving care at home? Can you describe what the healthcare staff do when they come to your home? Do you feel safe receiving care at home? Do you know how to contact healthcare if your general condition worsens?

The data collection period extended over several years due to challenges inherent in recruiting this frail older population. Some potential participants had reduced cognitive capacity or hearing impairments, making telephone interviews difficult to conduct. Additionally, two of the participating mobile care units were discontinued following post-COVID organisational changes, which further reduced the pool of eligible patients. These factors contributed to a slower and more prolonged recruitment process than initially anticipated.

### Data analysis

The analysis of the transcribed interviews was conducted according to a manifest conventional content analysis as described by Hsieh and Shannon (2005). All coding and categorisation were conducted manually using Microsoft Word (Microsoft Corporation, Redmond, WA, USA), which supported the organisation of meaning units, codes, and categories. Approximately 90 initial codes were generated during the first coding cycle, which gradually condensed into around 30 final codes before being organised into six subcategories and two main categories. In this manifest analysis, the focus is on identifying directly observable content. Hence, the analysis focuses on what the patients say by staying close to the data, using their own words or phrases, and describing the visible and obvious in the data. Four transcripts were carefully read by all authors independently to gain both depth and breadth in understanding the material. Next, units of meaning that reflect key concepts or themes within the text of the four transcripts were identified and marked. The initial coding process was discussed among the authors. Through this, CT created codes that reflected the units of meaning. CT coded the rest of the transcripts, and the next steps were also performed by CT but discussed with the other authors. The codes were sorted into subcategories based on how the different codes were related and linked to each other. The subcategories were utilised to arrange and cluster codes into significant groups, which served as the foundation for the emerging subcategories. Based on the relationships between the subcategories, they were then consolidated into a smaller number of categories. The research findings were enhanced by incorporating specific quotations. These chosen segments, taken directly from the original dataset, were subsequently translated into English. A summary of the steps in the analysis process is presented in Table III.

**Table III.** Summary of the steps in the analysis process.

**1. Immersion in Data** Data was read multiple times to understand the overall content and context. **2. Initial Coding** Exact words and phrases capturing key thoughts or concepts were highlighted through detailed, word-by-word analysis. **3. Developing Codes** Labels for codes were developed based on impressions and thoughts, reflecting key ideas and themes from the text. **4. Categorising Codes** Codes were sorted into broader categories based on their relationships and linkages, organising them into meaningful clusters. **5. Refining Categories** Categories were reviewed and adjusted to ensure they accurately represented the data, capturing the nuances and complexities of the text.

## Results

The findings portray patients’ experiences of being cared for by a mobile care unit. Two categories and six subcategories were identified. The categories were: “The home as a care setting”, and “Comprehensive patient-centred care”. Table IV shows the categories and subcategories of the analysis.

**Table IV.** Overview of the categories and subcategories.

Categories	Subcategories
The home as a care setting	A calming and safe Environment Everyday life continues The hospital is safer in the event of a serious illness
Comprehensive patient-centred care	Quality care relationship Advice and decision support Accessibility and assistance

### The home as a care setting

This category highlights the aspects of patients’ experiences of their home as a caring place, balancing the benefits of a familiar and quiet environment with the recognition of the hospital’s critical role in managing serious health issues. This category contains three subcategories: A calming and cafe environment, everyday life continues, the hospital is safer in the event of a serious illness.

### A calming and safe environment

The patients identified several key advantages of receiving care at home, primarily emphasising the tranquility and familiarity of the home environment. They noted that avoiding interactions with numerous healthcare professionals reduced the risk of infections. Additionally, patients appreciated the ability to remain at home, thereby avoiding the dramatic and stressful situations often encountered in hospitals, such as long waits and witnessing other patients in critical conditions. Another significant advantage highlighted by the patients was the elimination of the complicated logistics associated with travelling to and from healthcare facilities. They explained that planning and undertaking such trips could be both challenging and energy-consuming, especially when feeling weak or ill. Receiving care at home allowed them to focus on their health without the added burden of transportation. Prior to the establishment of the mobile care unit, patients were frequent visitors to hospitals and health centres.

*“You have everything in the home. It's the calmness that you're used to. It's much easier to be at home. If you're going away, it's so tiring, you have no energy left. So, at the hospital there is such stress on everything, and it has to go so fast and stuff like that. So that's the main advantage”—*(10).

### Everyday life continues

By being cared for by a mobile care unit, the patients were able to continue with their daily tasks and take care of their homes, which contributed to a sense of normality and independence. They could cook, clean, and take care of other household chores, giving them a sense of control over their everyday lives. One of the patients stated that gardening was fun and rewarding, which could be continued if care was provided at home. Having the home as a care setting allowed them to live a more normal life despite their illness, which they experienced as a great advantage.

*“If I'm still at home, I have good opportunities because I have to say that I'm not the one who sits on a chair all day and just looks straight out, but I've always been active for me, I have things that I need to do”* (1).

### The hospital is safer in the event of a serious illness

The patients described that the home was a safe environment, but only up to a certain degree of severity of the illness. When they became seriously ill, they preferred to call an ambulance so that they could be treated at the hospital. For conditions such as shortness of breath or chest pains, they needed help faster compared to what the mobile care could provide. Patients felt that the hospital offered a sense of security through the presence of medical staff around the clock and access to advanced equipment. There were also situations where the mobile care unit could not provide the necessary help, for example in the case of fractures that required X-rays or chest pain that required heart monitoring. In these cases, the hospital's resources were crucial for the patients to feel safe and receive the care they needed.

*“The mobile care unit is good, but it does not replace emergency care, When I get really sick I get fear of death, then I don't want to be at home”* (12).

## Comprehensive patient-centred care

This category highlights the patients’ experiences during their interactions with the mobile care unit, emphasising the healthcare professionals ´ attentiveness and the comprehensive support provided to the patients throughout their time in care. This category contains three subcategories: quality care relationship, advice and decision support, accessibility and assistance.

### Quality care relationship

The patients perceived that the healthcare professionals had plenty of time for their care, fostering a sense of security and attentiveness. They valued the thorough examinations and the collaborative approach to their care, which made the care situations relaxed and personal. The continuity of care, with healthcare professionals familiar with their medical history, was particularly valuable. This continuity ensured a comprehensive understanding of their medical conditions, further reinforcing the patients’ sense of being well cared for.

*“First of all, that you get the same physicians and nurses, they learn your medical picture. Much safer than when you go into the health centre, because then I get different physicians every single time”* (13).

Being able to remain at home and receiving care in a familiar environment significantly contributes to their sense of security and well-being. The patients felt that the healthcare professionals genuinely listened to them and took their needs and wishes seriously. They appreciated the individualised nature of the care, which allowed them to have greater influence over their own treatment. The personal contact and the continuous follow-up made them feel seen and heard, which was highly valued.

*“The nurse took her time, and they listen to you, come to your home, and really listen and you were able to ask questions”* (15).

### Advice and decision support

Patients reported significant relief from the burden of making decisions about temporary health deteriorations that might require hospitalisation. They expressed confidence in the healthcare professionals within the mobile care unit to provide appropriate guidance. Additionally, the opportunity to enquire about their health status and receive clear, informative responses enhanced their sense of security.

*“It’s a bit difficult when you constantly have some pressure in your chest and know that when should you seek care. But if you know that they come every week, you feel taken care of”* (11).

Patients valued the prompt assessment of their medical conditions, expressing confidence in the healthcare professionals' ability to accurately determine the urgency of their situations. This assurance facilitated timely staff intervention, when necessary, thereby enhancing their sense of security and care.

*“If I call and want them to come visit. They will come, so they will also make an assessment of course of how urgent it is. Should we go right away or wait an hour”* (12).

### Accessibility and assistance

The patients described several challenges and wishes regarding the care they received at home. This included that the mobile care units were not available outside office hours or at weekends, causing frustration, anxiety and uncertainty. Patients expressed a strong desire for mobile care units to be available around the clock, i.e., to provide support at any time of the day. Additionally, patients were unsure whether they could call for ailments other than those the mobile care unit usually dealt with, highlighting a need for clearer guidelines on when and how to seek help. To improve guidelines on when help could be sought, a few mobile care units provided a phone number for emergencies. Nevertheless, when the mobile care units closed for the day, patients were referred to an additional phone number for inpatient care.

*“You couldn't get in touch with this unit. Because it was the weekend. It would be good if it was available all the time”* (7).

Overall, they described that the mobile care units would need to do more advertising for themselves or show that they exist. Several of the patients believed that many people seek emergency medical care unnecessarily, which they observed when they were in the emergency department or were at the health centre. They described that it felt rewarding to help alleviate the burden on healthcare in general and the emergency department in particular.

*“I’d never heard of it until I got the offer here. I didn't know this existed. So there should be more marketing”* (12).

## Discussion

To the best of our knowledge, this is the first study to examine the patients’ experiences of being treated by a mobile care unit in Sweden. The study provides valuable insights into patients' experiences of this specialised form of in-home care. Patients reported several benefits of mobile care, including the comfort of a familiar environment and avoidance of hospital-related stress and disruption. They appreciated the convenience of not having to travel to healthcare facilities, especially when they were not feeling well. The mobile care unit reduced the need for frequent hospital visits, allowing patients to focus more on their recovery in a supportive environment. Mobile care enabled patients to live more normally despite their illness. Finally, the study showed that patients preferred home care for less severe conditions but chose the hospital for acute illnesses.

The current study showed that participants identified multiple advantages of receiving care in the home setting, notably the psychological comfort associated with a familiar environment and the reduction of stress and disruption typically linked to hospital-based care. These findings align with the studies by Bove et al. (2022) and Ko et al. (2023), which showed that patients valued the peace and comfort of being in their own homes. A systematic review of the effects of home care concluded that older adults often experienced an increased sense of security and comfort when care was provided at home, primarily because of the familiar environment and greater control over their daily routines (Boland et al., 2017). At the same time, this review emphasised that the benefit of receiving care frommobile care unitslargely depends on the availability of sufficient resources and support, such as well-functioning home care services, family assistance, or technical aids to ensure that mobile care is genuinely safe and comfortable in practice. The availability issues were also evident in our study, as mobile care units were not accessible outside office hours or on weekends, leading to frustration, anxiety, and uncertainty. McCormack & McCance also argue that access to services is crucial to supporting person-centred processes (McCormack & McCance, 2006). We therefore propose a single, integrated telephone line that patients can call. Accessibility via integrated telephone is a model that in recent triage studies has been shown to reduce unnecessary ambulance calls by up to a third, while improving the safety of callers (Chair et al., 2024; Roivainen et al., 2020). If the home is not adapted to meet the needs of older individuals, or if sufficient support is lacking, this can lead to increased insecurity and thus person-centred care will not be achieved to a sufficient extent.

Patients in this study highlighted the convenience of avoiding the logistical challenges of travelling to healthcare facilities, especially when feeling unwell. They appreciated being spared from the stress and disruptions of hospital settings, such as long waits and exposure to critical situations. The mobile care unit reduced their need for frequent hospital visits, allowing them to focus on recovery in a more supportive and stress-free environment. Previous research shows that advanced home care eliminated the need for emergency department visits, which patients found significantly relieving. They described emergency department visits as stressful, with long waiting times and a high-pressure environment (Jarling et al., 2022; Ko et al., 2023; Saenger et al., 2020). Patients in our study noted that having the same healthcare professionals visit them at home made it easier, as they didn't have to repeatedly explain their medical history. Within McCormack & McCance’s framework, continuity and relationship-building constitute core components of the “person-centred processes” that foster a sense of safety; our findings illustrate how these processes are enabled when the same care team repeatedly visits the patient’s home (McCormack & McCance, 2006). Jarling et al. (2022) also emphasises the importance of continuity, personal contact, and trusting interactions in creating a sense of security and well-being for patients. This suggests that mobile care can reduce the stress and logistical difficulties of hospital visits, allowing patients to concentrate on their recovery in a more relaxed and supportive environment. Asking patients how they want to receive care and involve them in decision making not only enhances their autonomy but also promotes improved well-being, while avoiding unnecessary effort from transport and movement. Through the lens of the McCormack & McCance’s framework these experiences can be interpreted as concrete manifestations of empowerment, whereby patients gain greater control over decision-making and care planning (McCormack & McCance, 2006).

Being cared for by a mobile care unit reduced the need for frequent hospital visits and thereby allowed patients to maintain their daily routines and manage their households, fostering a sense of normality and independence. They could engage in activities like cooking, cleaning, and gardening, which gave them control over their lives. This home-based care setting enabled them to live more normally despite their illness, which they found highly beneficial. Our findings align with the statements about the advantages of home-based care, such as maintaining daily routines and fostering a sense of normality and independence (Bove et al., 2022; Lin et al., 2024; Saenger et al., 2020). This suggests that involving mobile care units to assess and address the unique needs of older patients could enhance their ability to manage daily activities and improve their quality of life. As a complement it would also be important to develop individual care plans that include support for daily activities such as cooking, gardening and household chores. Finally, it can be valuable to allow patients to participate in scheduling visits from mobile care teams to ensure that the patients’ routines remain uninterrupted.

Patients described that the home could offer a safe environment with less severe conditions, but when the illness became more serious, they preferred hospital care. At a conceptual level, this reflects a central tension between the autonomy and comfort of home and the institutional safety associated with hospital-based monitoring. Patients viewed home as safe only for less severe conditions. When more severe symptoms occurred, they preferred hospital admission for rapid intervention and access to advanced diagnostics. Continuous presence of healthcare professionals and specialised equipment reinforced feelings of security. Mobile care was valued for managing minor issues but considered insufficient for urgent or complex needs. These findings highlight the need to clarify the limitations of mobile care and to ensure seamless transitions to emergency services when required. Regarding the safety of home-based care programmes, other studies show similar results, with some patients feeling less safe when dealing with acute illnesses (Bove et al., 2022; Levine et al., 2022). Bove et al. (2022) observed that while patients expressed appreciation for mobile care, concerns were raised regarding its perceived limitations in terms of response time and the availability of advanced medical interventions compared to hospital-based care. Previous studies also showed that patients associate mobile care with lower intensity of care and did not feel comfortable having strangers in their homes (Rossinot et al., 2019; Saenger et al., 2020; Wang et al., 2024). Drawing on the findings of this study and prior research, it appears that clear communication and regular follow-ups by mobile care teams may play a critical role in reducing patient anxiety by reinforcing the perception of continuous access to professional medical support in the event of health deterioration. By addressing concerns through improved education on the safety and effectiveness of mobile care, while ensuring strong support systems are in place, it is possible that we could boost patient confidence to engage with mobile care so that it can be used as a complement to traditional hospital care. By systematically mapping and cultivating a supportive care environment, consistent with the framework proposed by McCormack and McCance (McCormack & McCance, 2006), mobile home care services can be strategically developed to ensure the sustainability of person-centred care processes, even in the context of increasing patient volumes.

## Methodological considerations

There are limitations to this study. According to Mason (2010) there is no fixed rule for the number of participants in qualitative research, as the focus is on the depth and richness of the data rather than quantity. After 17 interviews, it became difficult to recruit more participants from the involved teams, and the research team stopped the data collection. Interview lengths ranged from 18 to 35 minutes except for one that lasted only 12 minutes. While Patton (2015) suggests that short interviews may limit the depth of data and miss nuances, Malterud (Malterud et al., 2016) argues that if participants provide rich information, less data can still be sufficient (i.e., information power). Despite some interviews being shorter than expected, the researchers felt the aim of the study was adequately answered and that the overall data was sufficient and varied. Given that the study participants were elderly and living with multiple chronic diseases, the interviews were carefully planned to ensure participants were alert and capable of engaging. To allow the reader to assess the trustworthiness of our findings, we have described the study setting and provided a detailed, transparent description of the analysis process. Credibility was strengthened through investigator triangulation, multiple readings of the transcripts, and peer debriefing within the research group. Dependability was supported by a systematic and clearly documented analytic process, in which coding decisions and category development were continuously recorded. Confirmability was enhanced through reflexive discussions and repeated returns to the data to ensure that interpretations were grounded in participants’ accounts rather than researchers’ preconceptions. The researchers have described the care model and the participants in the study to enhance transferability. Patton (2015) emphasises that transferability is a judgement made by the reader based on the details the researcher provides about the context and participants. Despite these limitations, we consider this study crucial for gaining insights into patients' experiences with mobile care.

## Conclusion

Patients receiving mobile home care appreciated the person-centred approach, tranquility, and comfort, as well as the reduced infection risks and logistical challenges associated with hospital visits. They valued the ability to maintain daily routines and independence, which significantly enhanced their quality of life. However, they expressed concerns about the effectiveness and safety of mobile care for more severe conditions, preferring hospital care for its advanced medical resources and staff availability around the clock. For mobile care to be effective, it is crucial to address the unique needs of older individuals, ensuring adequate support and involving them in care decisions. By fostering autonomy and minimising barriers, mobile care units can further improve daily functioning. Clear communication, regular follow-ups, and personalised care plans can help ensure continuity of care, boosting patient confidence and enhancing both safety and effectiveness of home-based services.

## Data Availability

The datasets used and/or analysed during the current study are available from the corresponding author on reasonable request.
